# The Role of Chemerin in Upper Gastrointestinal Cancer

**DOI:** 10.3390/metabo14110599

**Published:** 2024-11-07

**Authors:** Adam Mylonakis, Maximos Frountzas, Irene Lidoriki, Alexandros Kozadinos, Areti Kalfoutzou, Eva Karanikki, Iliana Tsikrikou, Maria Kyriakidou, Dimitrios Theodorou, Konstantinos G. Toutouzas, Dimitrios Schizas

**Affiliations:** 1 First Department of Surgery, Laikon General Hospital, National and Kapodistrian University of Athens, 11527 Athens, Greece; adam.mylonakis@med.uoa.gr (A.M.); akozadinos@med.uoa.gr (A.K.); il.tsikrikou@gmail.com (I.T.); mkyriakidou@nyc.gr (M.K.); dschizas@med.uoa.gr (D.S.); 2 First Propaedeutic Department of Surgery, Hippocration General Hospital, National and Kapodistrian University of Athens, 11527 Athens, Greece; froumax@med.uoa.gr (M.F.); dimitheod@netscape.net (D.T.); tousur@med.uoa.gr (K.G.T.); 3 Department of Environmental, Occupational Medicine and Epidemiology, Harvard T.H. Chan School of Public Health, Boston, MA 02139, USA; 4 Department of Occupational Medicine, Cambridge Health Alliance, Harvard Medical School, Cambridge, MA 02139, USA; 5 Department of Oncology, 251 Air Force General Hospital, 11525 Athens, Greece

**Keywords:** chemerin, gastric cancer, esophageal cancer, obesity, biomarker

## Abstract

**Background/Objectives**: Chemerin, which is a multifunctional cytokine and adipokine, has been implicated in inflammatory and metabolic processes and might play a role in upper gastrointestinal (GI) malignancies, particularly gastric and esophageal cancer. The aim of this review is to explore the role of chemerin in the pathophysiology of upper GI cancers, as well as its potential as a biomarker for early detection and as a therapeutic target. **Methods**: A comprehensive review of recent studies about chemerin’s biochemical properties and interaction with its receptors, as well as its effects on inflammatory responses, immune regulation, and metabolic processes, was conducted. The clinical implications of chemerin for gastric and esophageal cancer were analyzed, whereas the potential therapeutic strategies targeting chemerin were discussed. **Results**: Elevated chemerin levels are associated with poor prognosis in gastric cancer and promote invasiveness and metastasis in esophageal cancer. Chemerin receptor antagonists show promising results in inhibiting cancer cell migration, invasion, and progression. **Conclusions**: Chemerin could represent a valuable prognostic biomarker and therapeutic target for upper GI cancers. Future observational studies should validate its clinical applications and investigate the efficacy of chemerin inhibitors as potential therapeutic targets.

## 1. Introduction

Chemerin, also known as retinoic acid receptor responder 2 (RARRES2), is a 163-amino-acid protein, first identified in psoriatic skin lesions as a tazarotene-induced gene 2 protein (TIG2), that, a few years later, was described as a natural ligand for chemokine-like receptor 1 (CMKLR1, previously known as ChemR23) [1]. Chemerin is widely found in endocrine tissue, muscle, skin, lung, liver, and pancreas [2].

Upper gastrointestinal (GI) cancers, specifically gastric and esophageal cancer, are significant global health burdens, particularly due to their high morbidity and mortality. These malignancies are closely linked with various modifiable risk factors, including dietary habits, smoking, and notably, obesity. Obesity, a major public health issue worldwide, is a known risk factor for several types of cancer, including those of the upper GI tract. The interplay between obesity and upper GI cancers is mediated by complex pathophysiologic mechanisms involving inflammation, insulin resistance, and hormonal disruption. This connection emphasizes the need for advancing preventive strategies, early detection, and effective therapeutic approaches. Research focused on biomarkers such as chemerin, which is implicated in both metabolic regulation and inflammation, could provide critical insights into the pathophysiology of upper GI cancers and offer new opportunities for targeted therapies, potentially improving outcomes for cancer patients.

The involvement of chemerin in upper GI cancers is of particular interest due to the association of these cancers with inflammatory and metabolic processes. The dual role of chemerin in modulating immune responses and metabolic pathways could potentially demonstrate an association between obesity, which is a known risk factor for upper GI cancers, and tumor development [3]. This makes chemerin a valuable biomarker for early detection but also a promising novel target for therapeutic intervention. By targeting chemerin, the inflammatory and metabolic cascades, which contribute to tumor growth, might be disrupted. Therefore, investigating the metabolic pathways of chemerin regarding upper GI tumor interaction is critical to highlight its potential role in affecting disease progression and enhancing therapeutic outcomes for patients with such cancers.

## 2. Chemerin: Biochemistry and General Functions

### 2.1. Biochemical Properties, Synthesis, Main Receptors, and Signaling Pathways of Chemerin

Chemerin is initially synthesized as a 163-amino-acid precursor, preprochemerin, which undergoes proteolytic cleavage to remove a 20-amino-acid N-terminal signal peptide, yielding a 143-amino-acid protein, known as prochemerin. This precursor protein is further processed by several serine and cysteine proteases, resulting in different bioactive isoforms, including chemerinS157, chemerinF156, and others. These isoforms differ in their affinity for the chemerin receptors, particularly CMKLR1, with chemerinS157 and chemerinF156 exhibiting the highest binding affinity. The proteolytic processing of chemerin regulates its biological activity and also determines its function in different tissues. For example, chemerin in adipose tissue is crucial for adipogenesis and energy metabolism, whereas in the skin, it is involved in inflammatory responses and antimicrobial defense [4,5].

Chemerin exerts its biological effects primarily by interacting with three G protein-coupled receptors (GPCRs): chemokine-like receptor 1 (CMKLR1), C-C chemokine receptor-like 2 (CCRL2), and G protein-coupled receptor 1 (GPR1). Each of these receptors plays a unique role in mediating chemerin’s physiological processes and functions across different tissues.

First of all, chemokine-like receptor 1 (CMKLR1), is the most well-recognized receptor for chemerin. It is expressed in a variety of tissues, including adipose tissue, immune cells, and endothelial cells [6,7]. Upon binding to chemerin, CMKLR1 activates several intracellular signaling pathways. These include the mobilization of Ca^2+^, inhibition of Cyclic Adenosine monophosphate (cAMP) accumulation, and phosphorylation of Mitogen-Activated Protein Kinases (MAPKs), such as p42/p44 and p38 MAPK. These signaling events lead to diverse cellular responses, including chemotaxis, adipogenesis, and the modulation of inflammatory processes [7,8].

On the other hand, C-C chemokine receptor-like 2 (CCRL2) does not induce traditional GPCR signaling pathways such as Ca^2+^ mobilization or cAMP inhibition upon chemerin binding. Instead, CCRL2 acts as a non-signaling “decoy” receptor that binds chemerin without initiating signal transduction [9]. This receptor is primarily involved in presenting chemerin to cells expressing CMKLR1, thereby enhancing local chemerin concentrations and facilitating CMKLR1-mediated responses [9,10]. CCRL2 is expressed in leukocytes, endothelial cells, and other immune cells; thus, its role is particularly significant in inflammatory conditions [10].

In addition, G protein-coupled receptor 1 (GPR1), the third known receptor for chemerin, shares structural similarities with CMKLR1 but exhibits distinct functional properties. Although GPR1 binds chemerin with high affinity, it induces only weak Ca^2+^ mobilization compared to CMKLR1. The primary signaling mechanism of GPR1 involves arrestin’s recruitment rather than traditional G protein-mediated pathways [11]. This arrestin-mediated signaling contrasts with the rapid, second-messenger-driven responses typical of G protein activation. Instead, it leads to more sustained cellular responses, such as receptor internalization and the activation of alternative pathways including MAPKs, which regulate long-term cellular processes [12]. GPR1 is predominantly expressed in the central nervous system and certain peripheral tissues, suggesting a role in modulating neuroendocrine and metabolic functions [13,14]. The specific physiological and pathological roles of GPR1 are still under investigation, but its distinct expression pattern indicates functions that are different from those mediated by CMKLR1 and CCRL2 [14].

**Figure 1:**
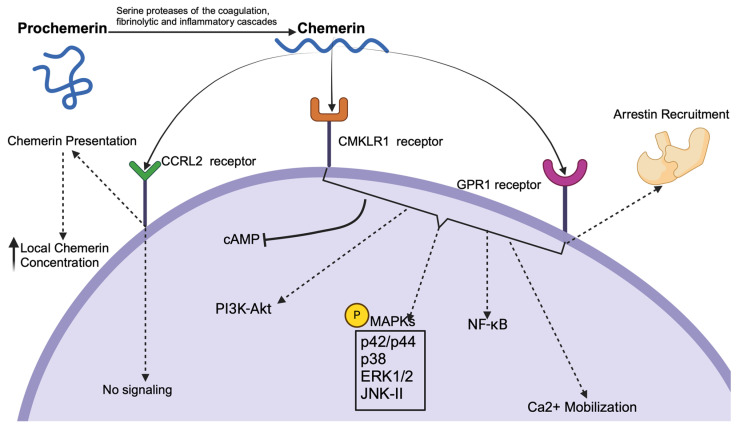
The signaling pathways activated by chemerin through its receptors CMKLR1, GPR1, and CCRL2. Key components include CMKLR1: Chemokine-like Receptor 1, GPR1: G Protein-Coupled Receptor 1, CCRL2: C-C Chemokine Receptor-Like 2, MAPK: Mitogen-Activated Protein Kinase, cAMP: cyclic Adenosine Monophosphate, ERK: Extracellular Signal-Regulated Kinase, JNK: c-Jun N-terminal Kinase), NF-κB: Nuclear factor kappa-light-chain-enhancer of activated B cells, and PI3K-Akt: Phosphoinositide 3-kinase-Akt (Created in BioRender.com on 8 October 2024).

Overall, the interaction of chemerin with its three receptors, each inducing distinct responses in different systems, underscores its multifaceted role in regulating immune responses, inflammation, adipogenesis, and various metabolic processes. A summarized overview of the signaling pathways associated with chemerin, including its activation, interaction with receptors, and downstream signaling effects, is presented in Figure 1.

### 2.2. Main Chemerin Functions

#### 2.2.1. Inflammatory Response

Chemerin is intricately involved in the inflammatory response, acting through its receptor CMKLR1 to recruit neutrophils, macrophages, and natural killer cells, thereby linking innate and adaptive immunity [15]. Elevated chemerin levels are observed in various inflammatory conditions, such as obesity [16], rheumatoid arthritis [17], psoriasis [18], and sepsis [19], correlating with disease severity. Under these circumstances, chemerin can modulate inflammation by inducing the production of pro-inflammatory cytokines, such as Tumor Necrosis Factor Alpha (TNF-α) and Interleukin 6 (IL-6) through the Extracellular Signal-regulated Kinases 1/2 (ERK1/2), p38 MAPK, and Phosphoinositide 3-kinase-Akt (PI3K-Akt) signaling pathways [20]. For instance, in diabetic nephropathy, the chemerin/CMKLR1 axis promotes inflammation in glomerular endothelial cells, further exacerbating the disease by increasing TNF-α, IL-6, and Interleukin 8 (IL-8) levels [21]. Similarly, in intervertebral disc degeneration, chemerin facilitates the activation of the Nuclear factor kappa-light-chain-enhancer of activated B cells (NF-κB) signaling pathway, leading to matrix degradation and cell aging [22]. Interestingly, chemerin exhibits dual roles, showing anti-inflammatory effects in some contexts. For example, it can inhibit neutrophil adhesion and promote the clearance of inflammatory cells from mucosal surfaces, as demonstrated in studies on epithelial cells with the chemerin/resolvin E1 anti-adhesion phenotype [23]. Additionally, synthetic chemerin-derived peptides have been shown to suppress inflammation by inhibiting macrophage activation and reducing neutrophil and monocyte recruitment in in vitro models of peritonitis [24]. Thus, chemerin’s involvement in inflammation is multifaceted, acting both as a pro-inflammatory and anti-inflammatory mediator, depending on its interaction with the particular tissue environment and the specific inflammatory conditions.

#### 2.2.2. Immune Regulation

Chemerin plays a critical role in immune regulation by modulating the activities of various immune cells and signaling pathways. Through its receptor CMKLR1, chemerin not only recruits immune cells, such as dendritic cells and macrophages to inflammation sites but also influences their activation and maturation [15]. This recruitment and activation are essential for the initiation and resolution of immune responses. Chemerin also impacts the differentiation and function of T cells, thereby linking innate and adaptive immunity [25]. For instance, chemerin can promote the development of regulatory T cells, which are vital for maintaining immune tolerance and preventing autoimmune responses [26]. Furthermore, chemerin influences the balance between pro-inflammatory and anti-inflammatory cytokines, modulating the immune response’s intensity and duration [3]. By regulating the immune cell infiltration and cytokine environment, chemerin ensures a balanced immune response, which is crucial for effective pathogen defense and tissue homeostasis.

#### 2.2.3. Metabolic Processes

Chemerin plays a central role in metabolic processes, particularly in lipid and glucose metabolism, and its dysregulation has substantial implications for metabolic disorders and possibly carcinogenesis. Primarily secreted by adipose tissue, chemerin influences lipid metabolism by promoting adipocyte differentiation and enhancing lipid storage in both adipose and non-adipose tissues, leading to an accumulation of triglycerides [27,28]. This dysregulated lipid metabolism often results in imbalances in lipid profiles, a common feature of obesity and metabolic syndrome [29]. Additionally, chemerin’s interference with insulin signaling disrupts glucose metabolism, contributing to insulin resistance, a hallmark of type 2 diabetes and related metabolic conditions. As insulin resistance progresses, glucose uptake decreases, promoting hyperglycemia and worsening metabolic dysfunction [30]

Chemerin’s metabolic effects also extend to hepatic lipid accumulation, where elevated chemerin levels are linked to non-alcoholic fatty liver disease (NAFLD) and its progression to non-alcoholic steatohepatitis (NASH) [31]. In the liver, chemerin stimulates pathways that lead to passive lipid buildup via receptors such as GPR1, contributing to a fatty liver phenotype characteristic of metabolic syndrome [32]. Over time, the liver’s capacity to handle this lipid influx diminishes, causing a cascade of metabolic disturbances that exacerbate conditions such as NAFLD. Moreover, chemerin’s impact on lipid and glucose homeostasis can impair pancreatic function, where prolonged lipid toxicity and glucose imbalances stress β-cells, further driving cellular dysfunction and advancing the metabolic deterioration [33].

In addition to its role in adipose and liver tissues, chemerin levels correlate with metabolic syndrome markers, including body mass index (BMI), triglyceride levels, and blood pressure [33]. Elevated chemerin levels contribute to metabolic dysfunctions observed in obesity, including insulin resistance in both the liver and skeletal muscle. By acting as a chemotactic factor, chemerin recruits immune cells to adipose tissue, perpetuating low-grade chronic inflammation, which, alongside metabolic dysregulation, may set the stage for carcinogenesis, particularly in the upper gastrointestinal (GI) tract [34]. Studies suggest that chemerin’s influence on lipid storage and glucose metabolism may create an environment conducive to DNA damage and tumor growth, with increased lipid availability supporting cancer cell proliferation in gastric and esophageal tissues [35]. Consequently, chemerin’s regulatory role in energy balance and lipid storage highlights its potential as both a biomarker and a therapeutic target for managing metabolic syndrome and reducing cancer risk associated with metabolic disorders.

## 3. Chemerin in Esophageal Cancer

**Figure 2:**
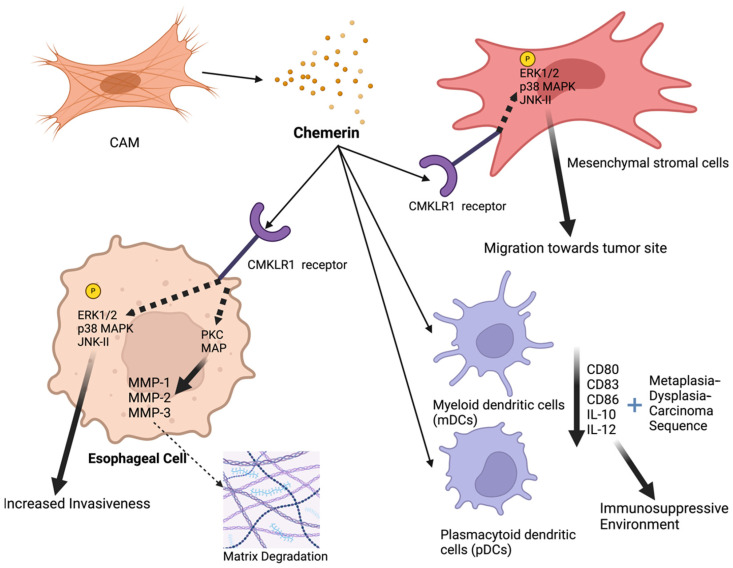
The mechanisms of the tumor-promoting effects of chemerin in esophageal cancer: Chemerin, released from cancer-associated myofibroblasts (CAMs), acts on CMKLR1 receptors present on esophageal cancer cells and mesenchymal stromal cells (MSCs), activating several intracellular signaling pathways, including ERK1/2, p38 MAPK, JNK-II, and PKC. This signaling leads to the increased expression and activity of Matrix Metalloproteinases (MMP-1, MMP-2, MMP-3), resulting in matrix degradation and enhanced invasiveness of esophageal cancer cells. Chemerin also recruits MSCs to the tumor site, promoting further tumor progression. Additionally, chemerin influences the tumor microenvironment by recruiting myeloid dendritic cells (mDCs) and plasmacytoid dendritic cells (pDCs), which display reduced expression of antigen-presenting molecules (CD80, CD83, CD86) and altered cytokine secretion (increased IL-10, decreased IL-12), creating an immunosuppressive environment. These effects collectively contribute to the progression of esophageal cancer through increased tumor cell migration, invasion, and immune evasion. Key components include ECM (extracellular matrix), ERK1/2 (extracellular signal-regulated kinase 1/2), IL-10 (interleukin 10), IL-12 (interleukin 12), MAPK (Mitogen-Activated Protein kinase), PKC (Protein Kinase C), and MMPs (Matrix Metalloproteinases). (Created in BioRender.com on 10 September 2024).

Esophageal cancer, one of the most lethal cancers globally, is known for its aggressive nature and poor prognosis [36]. It ranks as the sixth leading cause of cancer-related deaths worldwide, and its prevalence is rising, particularly in developed countries, where adenocarcinoma has become more common than squamous cell carcinoma [37]. There are two main types of esophageal cancer: squamous cell carcinoma, typically linked to smoking, alcohol consumption, and a low intake of fruits and vegetables [38]; and adenocarcinoma, often associated with gastroesophageal reflux disease, obesity, and Barrett’s esophagus [37]. Other risk factors include hot beverage consumption, poor oral health, and low socioeconomic status [36]. The role of chemerin in esophageal cancer is still under investigation, with limited data pointing to an oncogenic role of chemerin for this malignancy, as shown in Figure 2.

The study by Somja et al. explored the role of dendritic cells (DCs) in the progression of Barrett’s esophagus (BE) to esophageal adenocarcinoma (EAC), emphasizing the role of chemerin [39]. The researchers found that both myeloid dendritic cells (mDCs) and plasmacytoid dendritic cells (pDCs) are recruited during the metaplasia–dysplasia–carcinoma (MDC) sequence of BE, and chemo-attractants, such as chemerin and macrophage inflammatory protein 3a (MIP3a), are responsible for this recruitment. Chemerin is increasingly being expressed throughout the disease progression. In BE, chemerin expression was weak, but it was increased significantly in low-grade dysplasia (LGBE), high-grade dysplasia (HGBE), and EAC. Specifically, chemerin expression scores increased from 1 in BE to 3 in EAC (*p* < 0.001), and this increased expression was correlated with the recruitment of mDCs and pDCs. Functionally, mDCs that were co-cultured with BE and EAC cell lines displayed reduced expression of antigen-presenting molecules such as CD80, CD83, and CD86. Additionally, these mDCs showed increased secretion of the anti-inflammatory cytokine IL-10 and decreased secretion of the pro-inflammatory cytokine IL-12, further promoting an immunosuppressive tumor environment.

A study by Kumar et al. investigated the role of chemerin in recruiting Mesenchymal Stromal Cells (MSCs) to the tumor microenvironment of esophageal squamous cell carcinoma (ESCC) [40]. This study demonstrated that chemerin is significantly overexpressed in Cancer-Associated Myofibroblasts (CAMs) compared to adjacent tissue myofibroblasts (ATMs). Conditioned media (CM) from CAMs increased MSC migration by 2.1-fold compared to ATM-CM. This effect was significantly reduced by chemerin-neutralizing antibody, which decreased migration by 60%, as well as chemerin siRNA, ChemR23 siRNA, and the ChemR23 receptor antagonist CCX832, which inhibited MSC migration by 50%. The authors demonstrated that chemerin stimulated MSC migration by activating several signaling pathways, including the phosphorylation of p42/44, p38 and c-Jun NH2-Terminal Kinase 2 (JNK2) kinases. Inhibitors of these kinases and Protein Kinase C (PKC) reversed chemerin-stimulated MSC migration. In a xenograft model with OE21 esophageal cancer cells and CAMs, MSC migration was increased by 75% when CAMs were present, and this effect was inhibited by CCX832 by 70%, showing both in vitro and in vivo in a xenograft model that CCX832 inhibits MSC migration in response to CAMs.

A follow-up study conducted by Kumar et al. explored the role of chemerin and its receptor ChemR23 in promoting the invasion of squamous esophageal cancer (OSC) cells [41]. The study confirmed the expression of ChemR23 in OSC tissues and the OSC cell line OE21 through immunohistochemistry. Functional assays revealed that chemerin significantly enhances the migration, invasion, and proliferation of OE21 cells in vitro. Additionally, conditioned media (CM) from CAMs, which is rich in chemerin, markedly increased OE21 cell invasion, an effect that was significantly reduced by chemerin immuno-neutralization, the ChemR23 antagonist CCX832, and chemerin-specific Small interfering RNA (siRNA). The study further investigated the mechanisms behind chemerin’s role in OSC cell invasion. It was found that chemerin increased the expression and activity of several Matrix Metalloproteinases (MMPs), particularly MMP-1, MMP-2, and MMP-3, in OE21 cells, facilitating cancer cell invasion. The induction of MMPs by chemerin was mediated through Protein Kinase C (PKC) and Mitogen-Activated Protein (MAP) kinase pathways, as inhibitors of these pathways significantly reduced MMP expression. The data suggested that chemerin released by CAMs acts on ChemR23 expressed on OSC cells, promoting an aggressive invasive phenotype via the upregulation of MMPs.

## 4. Chemerin in Gastric Cancer

**Figure 3:**
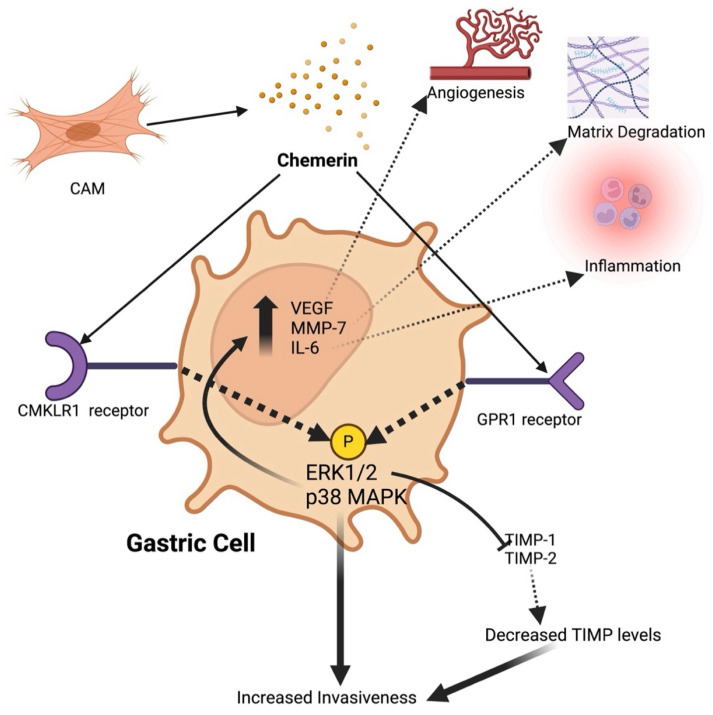
The mechanisms of the tumor-promoting effects of chemerin in gastric cancer: Chemerin is released from cancer-associated myofibroblasts (CAMs) and acts on CMKLR1 and GPR1 receptors present on gastric carcinoma cells to activate several intracellular signaling pathways. Functionally, this signaling leads to the increased expression of pro-invasive genes such as VEGF, MMP-7, and IL-6; reduced secretion of tissue inhibitors of metalloproteinases (TIMP-1 and TIMP-2); and enhanced production of Matrix Metalloproteinases (MMPs). These effects collectively result in the migration and invasion of tumor cells and tumor cell transformation resembling an epithelial-to-mesenchymal transformation (EMT). Additionally, chemerin signaling promotes angiogenesis, matrix degradation, and inflammation, contributing to the tumor-promoting environment. Key components include ERK1/2 (extracellular signal-regulated kinase 1/2), IL-6 (interleukin 6), MAPK (Mitogen-Activated Protein kinase), PKC (Protein Kinase C), TIMP (tissue inhibitors of metalloproteinases), and VEGF (Vascular Endothelial Growth Factor). (Created in BioRender.com on 10 September 2024).

Gastric cancer, one of the most common and deadly cancers worldwide, is characterized by its high incidence and poor prognosis. It ranks as the fifth most common cancer and the third leading cause of cancer-related deaths globally, with over 1 million new cases diagnosed annually [42,43]. The prevalence of gastric cancer is particularly high in Eastern Asia, Eastern Europe, and South America, with significant variations in histological subtypes and risk factors between different geographical regions [44]. Gastric cancer is primarily classified into two main types: intestinal and diffuse. Intestinal-type gastric cancer is often associated with chronic infection by Helicobacter pylori, dietary factors such as high salt intake, and smoking [45]. On the other hand, diffuse-type gastric cancer is linked to genetic predispositions and is less influenced by environmental factors [46]. Other risk factors for gastric cancer include obesity, Epstein-Barr virus infection, and pernicious anemia [47]. The prognosis for gastric cancer is poor, with 1-year and 5-year survival rates varying significantly based on the stage at diagnosis. The 5-year survival rate is less than 10% when diagnosed at an advanced stage but can be as high as 85% if detected early [48]. Unlike esophageal cancer, where the role of chemerin is still under investigation, there are more data available on the role of chemerin in gastric cancer. Research indicates that chemerin might play a significant role in the progression and metastasis of gastric cancer, potentially serving as a biomarker for diagnosis and a target for therapeutic interventions as shown in Figure 3.

In their study, Zhang et al. evaluated the prognostic significance of preoperative plasma chemerin levels in gastric cancer patients [49]. Researchers recruited 196 gastric cancer patients and 196 age- and gender-matched healthy controls, measuring preoperative plasma chemerin levels. They found that gastric cancer patients had significantly higher plasma chemerin levels compared to healthy controls. Elevated chemerin levels were identified as an independent predictor of 5-year mortality (OR, 2.718) and adverse events (OR, 2.982), with high area under the ROC curve (AUC) values for both outcomes, indicating strong predictive capability. Furthermore, high chemerin levels were associated with shorter overall survival (HR, 1.788) and disease-free survival (HR, 2.016). The study concluded that high plasma chemerin levels correlated with poor prognosis in gastric cancer, making it a valuable prognostic biomarker. Patients with higher chemerin levels had increased risks of mortality and adverse events, as well as significantly shorter overall and disease-free survival intervals.

Wang et al. investigated the serum levels of chemerin in gastric cancer patients and its biological effects on gastric cancer cells [50]. The study included 36 gastric cancer patients and 40 healthy subjects, measuring serum chemerin levels before surgical resection of gastric cancer. The results showed significantly higher serum chemerin levels in gastric cancer patients compared to healthy subjects, while increased levels were associated with advanced clinical stages and non-intestinal-type gastric cancer. The study further revealed that serum chemerin levels were significantly higher in patients with stage II and stage III + IV gastric cancer compared to stage I and healthy subjects. Additionally, in vitro experiments demonstrated that chemerin increased the invasiveness of gastric cancer cells and induced the phosphorylation of p38 and ERK1/2 MAPKs, while it upregulated pro-invasive genes, such as VEGF, MMP-7, and IL-6. Notably, the inhibition of ERK1/2 phosphorylation abolished the pro-invasive effects of chemerin, highlighting a novel action of chemerin in promoting gastric cancer cell invasiveness. Chemerin exposure increased gastric cancer cell invasiveness without affecting cell proliferation, and this effect was mediated through the activation of the ERK1/2 MAPK pathway. Pharmacological inhibition of this pathway prevented chemerin-induced invasiveness and the upregulation of pro-invasive genes. These findings suggest that increased serum chemerin levels are associated with advanced stages and aggressive types of gastric cancer and that chemerin may serve as a potential target for therapeutic intervention in gastric cancer.

In another study, Kumar et al. investigated the role of chemerin and its receptors, CMKLR1 and GPR1, in promoting the migration and invasion of gastric cancer cells [35]. Immunohistochemical analysis of gastric cancer cells from 15 patients revealed high expression levels of CMKLR1 and GPR1 in nearly all cancer cells, with no evident difference between intestinal, diffuse, or mixed gastric cancers or TNM stage. Chemerin was found to be secreted by gastric cancer-associated myofibroblasts (CAMs) but not by gastric cancer cells. The study demonstrated that chemerin significantly increased the in vitro invasiveness of gastric cancer cells in Boyden chamber assays, with a 5-fold increase in migration at a chemerin concentration of 0.1 ng/mL, without affecting their proliferation. This effect was found to be mediated through the activation of the ERK1/2 MAPK pathway. The investigators demonstrate that both CMKLR1 and GPR1 are involved in these processes, by selectively inhibiting these receptors and observing a significant but not complete suppression of cancer cell migration. Furthermore, the authors suggest that chemerin promotes gastric cancer cell invasion partly by suppressing Tissue Inhibitors of Metalloproteinases TIMP-1 and TIMP-2. This was established by correlating the reduction in TIMP levels with increased cancer cell invasiveness, while supplementation with TIMP-1 (2.1 nM) and TIMP-2 (2.5 nM) significantly inhibited the chemerin-induced migration and invasion responses. These findings suggested that chemerin promotes gastric cancer cell invasion partly by suppressing TIMP-1 and TIMP-2, highlighting the potential role of chemerin receptor antagonists as therapeutic agents to inhibit gastric cancer progression.

## 5. Clinical Implications of Chemerin

### 5.1. Prognostic Value and Therapeutic Targeting of Chemerin and Its Receptors

Circulating chemerin levels have been shown to be significantly elevated in cancer patients compared to healthy controls, with a pooled standardized mean difference of 1.47 (95% CI: 1.03–1.90), suggesting a strong correlation between high chemerin levels and increased cancer risk [51]. This correlation has been established in gastric, non-small cell lung, breast, and ovarian cancers, where chemerin enhances cell migration, invasion, and angiogenesis, contributing to tumor progression [52,53]. Specifically, in gastric cancer, elevated serum chemerin levels are significantly higher compared to healthy controls and also correlate with advanced clinical stages, non-intestinal types of gastric cancer, and increased cellular invasiveness. Higher chemerin levels are associated with increased expression of VEGF, MMP-7, and IL-6, which promote tumor metastasis [50]. Moreover, chemerin levels serve as an independent predictor of 5-year mortality, overall survival (OS), and disease-free survival (DFS), underscoring its value as a prognostic biomarker in gastric cancer [49]. Overall, elevated chemerin levels are associated with advanced disease stages, metastasis, and poorer survival outcomes in GI cancers, making it a valuable prognostic biomarker for these malignancies.

Considering its role in promoting cancer cell invasiveness and progression, chemerin and its receptors represent potential therapeutic targets. Studies on the chemerin receptor antagonist CCX832 have shown promising results in inhibiting cancer cell invasion and progression [54]. In squamous esophageal cancer (OSC), the chemerin receptor ChemR23 is expressed in OSC cells, where chemerin stimulates invasion. CCX832 inhibits this invasion by reducing the activity of matrix metalloproteinases (MMPs), which are essential for cancer cell invasion [41]. In gastric cancer, chemerin promotes the migration and invasion of cancer cells through its receptors CMKLR1 and GPR1. CCX832 inhibits these effects by downregulating tissue inhibitors of metalloproteinases (TIMP-1 and TIMP-2), highlighting chemerin’s role in enhancing cancer cell invasiveness [39]. Furthermore, in esophageal squamous cancer, chemerin is overexpressed in cancer-associated myofibroblasts (CAMs). CCX832 inhibits the chemerin-induced recruitment of mesenchymal stromal cells (MSCs) to the tumor site, potentially delaying tumor progression by preventing the recruitment of supportive stromal cells [40]. These studies collectively highlight the potential role of CCX832 in reducing cancer cell invasion and progression by inhibiting the chemerin/ChemR23 signaling pathway, offering a promising therapeutic strategy for managing gastrointestinal cancers.

### 5.2. Integration into Current Clinical Practice

Chemerin-targeted therapies could enhance cancer treatment by complementing existing modalities such as chemotherapy, radiotherapy, and immunotherapy [55]. These therapies can improve treatment efficacy and reduce side effects by upregulating Phosphatase and Tensin Homolog (PTEN) and suppressing Programmed death-ligand 1 (PD-L1) in tumor cells, thereby boosting immune responses and recruiting NK and T cells to the tumor microenvironment [55,56]. Synthetic derivatives of chemerin, which are metabolically stable, can serve as precision carriers for chemotherapeutic agents against tumor cells [57]. Furthermore, combining chemerin with existing therapies can prevent chemotherapy-induced cachexia and enhance the immune clearance of tumor cells [58]. Overall, chemerin-targeted therapies represent a significant advancement in personalized cancer treatment, offering a comprehensive and potent approach to tumor suppression [59]. Combining chemerin receptor antagonists with standard chemotherapy could enhance the overall response rate by simultaneously targeting tumor cells and the tumor microenvironment, presenting an extremely promising and emerging therapeutic approach [60].

## 6. Future Directions and Research Needs

### 6.1. What Is Missing with Respect to Chemerin’s Role in Upper GI Cancers?

While substantial progress has been made in understanding the role of chemerin in upper gastrointestinal cancers, several critical gaps still remain. One significant gap is the lack of comprehensive data on chemerin levels and their association with surgical interventions or chemotherapy, which are the gold standard for esophageal and gastric cancer. Understanding how chemerin levels fluctuate in response to these treatments could provide valuable insights into its potential role as a biomarker for treatment efficacy and disease progression [49] Specifically, evaluating the changes in chemerin levels as a response to chemotherapy could be particularly valuable in assessing treatment effectiveness and guiding therapeutic adjustments [50].

Another critical gap is the need for detailed studies on chemerin levels in different tissues, including adipose tissue and cancer tissue. Both omental and subcutaneous fat can be used for these assessments, as these adipose tissue depots might provide valuable information on the local production and action of chemerin within the tumor microenvironment. Notably, studies have shown that chemerin mRNA expression is significantly higher in subcutaneous adipose tissue compared to visceral (omental) adipose tissue, indicating depot-specific differences in chemerin expression [61]. Additionally, it is unclear whether blood levels of chemerin are as reliable as tissue levels in reflecting disease status, which also highlights the need for comparative studies to determine the most accurate biomarkers for monitoring treatment efficacy and disease progression.

Furthermore, the correlation between cancer and the potential rise in chemerin levels due to obesity remains unclear, necessitating further research to elucidate this relationship and its implications for cancer progression and treatment [62,63] Conversely, investigating the relationship between chemerin levels and cachexia could uncover mechanisms by which chemerin influences muscle wasting and weight loss, which represent common complications in advanced cancer stages [58]. This information could lead to more personalized and precise treatment strategies, improving patient outcomes and optimizing therapeutic regimens.

Lastly, the association of chemerin levels with cancer stage, morbidity, and mortality, as well as patient cachexia and nutritional status, in upper GI cancer patients requires further investigation. Understanding these associations could provide crucial insights into the prognostic value of chemerin as a biomarker. Specifically, correlating chemerin levels with TNM staging could potentially identify the extent of cancer progression and metastasis [42,43]. Exploring the link between chemerin levels and morbidity and mortality rates may reveal its role in predicting patient outcomes and survival rates [49]. Such detailed studies could help in stratifying patients based on their risk profiles and tailoring personalized treatment approaches, ultimately improving clinical management and outcomes for upper GI cancer patients.

### 6.2. State-of-the-Art Assessment of Chemerin

Advancements in technology and methodology can significantly enhance the assessment of chemerin in both clinical and research settings. Standardized measurement techniques for novel adipokines, such as chemerin, are essential for reducing costs and promoting consistency across studies. The development of advanced ELISA kits and mass spectrometry-based assays can improve the accuracy and reliability of chemerin measurements in blood and tissue samples. Furthermore, the potential of saliva tests to become widely available in the future could offer a non-invasive and convenient method for chemerin assessment, broadening its application in routine clinical practice and large-scale epidemiological studies [64].

Additionally, adipose tissue biopsy could be explored as a viable method for assessing chemerin levels, particularly if adipose tissue proves to be a more reliable source than blood samples. Both omental and subcutaneous fat biopsies could be considered to determine the most effective site for chemerin assessment. This approach could provide a more direct measurement of chemerin’s role in metabolic and inflammatory processes within the tumor microenvironment [61,65].

Emerging imaging technologies, such as PET scans using radiolabeled chemerin analogs, could offer non-invasive methods to assess chemerin distribution and activity in vivo, as proposed for breast cancer [66]. These technologies could provide real-time insights into chemerin dynamics during disease progression and treatment, potentially guiding more effective therapeutic interventions.

### 6.3. Proposals for Future Research Focusing on Translational and Clinical Studies

Despite significant progress, our understanding of the role of chemerin in upper GI cancer remains limited, especially when compared to more well-studied adipokines. Translational studies should explore the mechanisms by which chemerin influences tumor biology and the tumor microenvironment. This could uncover new pathways and molecular targets for intervention, broadening the scope of therapeutic strategies available for upper gastrointestinal (GI) cancers.

Future research should aim to translate the current understanding of chemerin into clinical applications that can improve patient outcomes. Firstly, large-scale clinical trials are necessary to validate chemerin both as a prognostic biomarker and a therapeutic target. These trials should involve diverse patient populations to assess how chemerin levels influence treatment outcomes, particularly with chemotherapy and surgical interventions.

Additionally, initial studies should aim to establish the safety and efficacy of chemerin inhibitors using preclinical models. This includes using animal models to investigate the effects of chemerin inhibitors on tumor growth, metastasis, and responses to standard therapies in gastric and esophageal cancers. Furthermore, research should investigate the potential synergistic effects of combining chemerin inhibitors with other targeted therapies or immunotherapies, as well as conventional chemotherapy.

Collaborative efforts between academic institutions, clinical researchers, and the pharmaceutical industry are essential to accelerate the development and clinical application of chemerin-targeted therapies. Such collaborations can facilitate the sharing of resources, expertise, and data, ultimately advancing the field of cancer research and improving patient care.

In summary, addressing these research needs and leveraging emerging technologies can significantly enhance our understanding of chemerin’s role in upper GI cancers, paving the way for innovative and effective clinical applications.

## 7. Conclusions

Chemerin, a multifunctional cytokine and adipokine, plays a significant role in inflammatory and metabolic processes, making it a promising prognostic biomarker and therapeutic target for upper GI cancers. To fully realize its potential, comprehensive translational studies and large-scale clinical trials are needed to validate its clinical applications. Research should focus on understanding chemerin’s influence on tumor biology and exploring the efficacy of chemerin inhibitors, especially in combination with existing therapies. Collaborative efforts among researchers and the pharmaceutical industry are crucial to advancing chemerin-targeted therapies, ultimately improving outcomes for patients with upper GI cancer.

## Data Availability

Not applicable.
